# Anti-inflammatory efficacy of Berberine Nanomicelle for improvement of cerebral ischemia: formulation, characterization and evaluation in bilateral common carotid artery occlusion rat model

**DOI:** 10.1186/s40360-021-00525-7

**Published:** 2021-10-03

**Authors:** Roza Azadi, Seyyedeh Elaheh Mousavi, Negar Motakef Kazemi, Hasan Yousefi-Manesh, Seyed Mahdi Rezayat, Mahmoud Reza Jaafari

**Affiliations:** 1 Department of Medical Nanotechnology, Faculty of Advanced Sciences and Technology, Tehran Medical Sciences, Islamic Azad University, Tehran, Iran; 2 Department of Pharmacology, School of Medicine, Tehran University of Medical Sciences, Tehran, Iran; 3 Department of Medical Nanotechnology, School of Advanced Technologies in Medicine, Tehran University of Medical Sciences, Tehran, Iran; 4 Department of Pharmaceutical Nanotechnology, Mashhad University of Medical Sciences, Mashhad, Iran

**Keywords:** Berberine, BCCAO model, Cerebral ischemia, Nano formulation, Anti-inflammation

## Abstract

**Background:**

Berberine (BBR) is a plant alkaloid that possesses anti-inflammatory and anti-oxidant effects with low oral bioavailability. In this study, micelle formulation of BBR was investigated to improve therapeutic efficacy and examined its effect on the secretion of inflammatory cytokines in cerebral ischemia in the animal model.

**Material and methods:**

Nano formulation was prepared by thin-film hydration method, and characterized by particle size, zeta potential, morphology, encapsulation efficacy, and drug release in Simulated Gastric Fluid (SGF) and Simulated Intestine Fluid (SIF). Then, Wistar rats were pretreated with the drug (100 mg/kg) and nano-drug (25, 50, 75, 100 mg/kg) for 14 days. Then, on the fourteenth day, stroke induction was accomplished by Bilateral Common Carotid Artery Occlusion (BCCAO); after that, Tumor Necrosis Factor - Alpha (TNF-α), Interleukin – 1 Beta (IL-1ß), and Malondialdehyde (MDA) levels were measured in the supernatant of the whole brain, then the anti-inflammatory effect of BBR formulations was examined.

**Result and discussion:**

Micelles were successfully formed with appropriate characteristics and smaller sizes than 20 nm. The Poly Dispersity Index (PDI), zeta potential, encapsulation efficacy of micelles was 0.227, − 22 mV, 81%, respectively. Also, the stability of nano micelles was higher in SGF as compared to SIF. Our outcomes of TNF-a, IL-1B, and MDA evaluation show a significant ameliorating effect of the Berberine (BBR) and BBR-loaded micelles in pretreated groups.

**Conclusion:**

Our experimental data show that pretreated groups in different doses (nano BBR 100, 75, 50 mg/kg, and BBR 100 mg/kg) successfully showed decreased levels of the inflammatory factors in cerebral ischemia compared with the stroke group and pretreated group with nano BBR in the dose of 25 mg/kg. Nano BBR formulation with a lower dose can be a better candidate than conventional BBR formulation to reduce oxidative and inflammatory factors in cerebral ischemia. Therefore, BBR-loaded micelle formulation could be a promising protective agent on cerebral ischemia.

**Supplementary Information:**

The online version contains supplementary material available at 10.1186/s40360-021-00525-7.

## Introduction

Many studies have been done on cerebral ischemia and all of them have confirmed its morbidity and mortality worldwide [1]. Cerebral ischemia occurs due to a blockage in the carotid arteries, which results in damage to brain tissue. Results indicate factors such as oxygen-free radicals and inflammatory cytokines (such as TNF-α, IL-1ß) and Malondialdehyde (MDA) as a marker of oxidative stress that plays an important role in damaging brain tissue and neurons due to cerebral ischemia [2–4]. There is evidence that TNF-a and IL-1B levels in the brain increase many-fold (up to 40 or 60 times) during the first 24 h after inducing stroke [5]. Therefore, it is necessary to recognize safe compounds with high and effective therapeutic efficacy in reducing inflammation during the stroke.

Berberine (BBR), an isoquinoline alkaloid extracted from Coptidis rhizome and Cortex Phellodendron, is one of the herbal and safe compounds (C_20_H_18_NO_4_^+^) which exhibits anti-oxidative and anti-inflammatory effects on brain diseases such as ischemia [6–9], Alzheimer [10], and tumors [11]. Also, it has been reported that BBR possesses effects of antibacterial [12], Antioxidant [13], antiviral [14], and anticancer [15]. However, the therapeutics effectiveness of BBR is restricted due to its hydrophobic nature and poor water solubility [16]. Pharmacokinetic studies of BBR in animals indicate that BBR has low plasma concentration for 48 h because of its poor absorption rate in the gut wall about 33.6% within 1 h [17]. Also, it is metabolized rapidly in the liver that is the main metabolic site in the body, and after BBR absorption, the clearance of BBR from blood is fast. In the rat’s body, it is quickly transferred to the liver and bile through active transportation, then rapidly will be biotransformed [18, 19]. BBR has metabolized in the rat liver via phase I demethylation and phase II glucuronidation, then apparently excreted through the duodenum in bile [17]. Current findings indicate that the toxicity of BBR is mainly associated with intravenous administration.

Also, according to results in the drug bank, the BBR melting point is 145 °C, its water solubility is 0.000354 mg/mL, Log P is − 1.3, and bioavailability is 1 [20]. Therefore, to overcome these challenges, we need a novel formulation in the drug delivery system to improve drug solubility and efficiency.

As mentioned above, mucus on the gut wall is a barrier to the absorption of hydrophobic particles. So, applying nanotechnology and nanocarriers can be an effective way to overcome this challenge. Various studies were accomplished on nanocarriers for BBR delivery with an approach to the improvement and treatment of different types of disease. Some inorganic and organic nanocarriers used for BBR including gold nanoparticles, magnetic mesoporous nanoparticles [21], polymers [22], lipid-based [23], and so forth. Also, plentiful studies were done on the therapeutic effects of BBR on stroke ischemia, but none of them used nano-formulation. For instance, increased bioavailable BBR attenuated cerebral ischemia/reperfusion (I/R) injury in rats [24]. Research by Pishva and et al. showed the effect of berberine nano micelle on inflammatory mediators for the improvement of cirrhotic cardiomyopathy in rats. It has been recognized that nano micelle berberine compared with berberine possesses a better anti-inflammatory potential in cirrhotic injuries [25].

Lately, the hydrophobic drugs encapsulating micelles as nanocarriers have attracted much attention for drug delivery applications [26]. Micelles are amphiphilic colloidal structures with particle diameters from 5 to 100 nm range. The core of the micelle is formed by the hydrophobic fragments of amphiphilic molecules, whereas the micelle’s shell consists of hydrophilic micellar molecule fragments [27]. Micelles possess advantages for oral drug delivery, such as the ability to solubilize hydrophobic drugs due to their unique structure (core-shell) resulting in enhancing drug effectiveness without change or disruption in drug formulation [28–31]. So, the design of the drug delivery system based on micelles could show promising performances in oral administration by providing a high level of therapy.

The present study aimed to develop the drug formulation with an approach to nanoencapsulation and evaluation of its effect on the secretion of inflammatory mediators in stroke ischemia in the rat.

We tried focusing on the preparation of nano formulation by thin-film hydration method and its characterization, then after inducing stroke, levels of inflammation and oxidative factors (TNF-α, IL-1ß, MDA) were measured in brain tissue. Thus, the aim of this study was to preparation and comparison of BBR and BBR-loaded micelle formulations and survey their anti-inflammatory effects on cerebral ischemia-induced with Bilateral Common Carotid Artery Occlusion (BCCAO) model in the rats.

## Material and methods

Deoxycholate and BBR (98% purity) were purchased from Sigma. Ketamine hydrochloride and xylazine were from Alfasan (Holland). The chemical used including NaOH, KH_2_PO_4_, NaCl, HCl, and other materials and solvents were analytical grades. Equipment was used including rotary (Buchi, Switzerland), centrifuge, filter amicon 3000 KD, and spectrophotometer UV-Vis (UV-160 A, SHIMADZU – Japan).

### Preparation of BBR-loaded micelle solution

The Thin-film hydration method was used for the preparation of BBR-loaded micelle solution [32]. Briefly, in a round-bottom flask BBR (1% w/w) and deoxycholate (49% w/w) as the surfactant, was mixed with a minimum amount of methanol as an organic solvent. The rotary evaporator was applied for more than 2 h to evaporate solvent under reduced pressure and to deposit thin film. Then, in the hydration phase, deionized water (50% w/w) was added to it, and bain-marie was applied to 50 °C while stirring was done for 20 min at the same temperature until a clear solution was obtained that was light yellow.

### Characterization

Dynamic light scattering reported size and size distribution, PDI, Zeta potential of nano micelles (Malvern Instruments, UK). The transmission electron microscope (TEM) was applied to show the size of nano micelle formulations (PHILIPS CM 300, Netherlands).

In transmission electron microscope (TEM), samples were diluted in ethanol and placed under dispersion for 20 min. Then, nano micelles (1 drop) are deposited onto a carbon-coated copper grid. After dried, the samples viewed and photographed with a Phillips cm300 electron microscope. For the determination of encapsulation efficiency, an indirect method was used. Briefly, after centrifugation of micelles at 4000×g for 30 min, the concentration of BBR in the samples was determined by spectroscopy UV-Vis in λ max = 348 nm (UV-160 A, SHIMADZU – Japan). The drug encapsulation efficacy was calculated by the following equations [33]:

$$ EE\%=\frac{drug\ concentration\ post- filterate\ drug}{initial\ concentration\ of\ drug}\times 100 $$

### Drug release study

The Release pattern of BBR from nano micelles was investigated in SGF and SIF. To prepare the SGF, 246 μL HCl and 200 mg NaCl were added to 60 ml deionized water (DW), and pH was adjusted at 2, then the final volume was filled to 100 mL with DW. Also, SIF was prepared by dissolving 680 mg of KH_2_PO_4_ and 61.6 mg of NaOH in 60 mL DW, and pH was adjusted at 6.5. Then the final volume was filled to 100 mL with DW. Samples were diluted in a ratio of 1:10 in SIF and SGF and incubated at 37 °C. Then sampling was carried out at time points 0, 30 min, 1, 2, 4, 8, 24 h [34]. Then the determination of BBR was accomplished by spectroscopy UV-Vis in λ max = 348 nm (UV-160 A, SHIMADZU – Japan).

### Animal groups and ethical considerations

In this project, 96 adult male Wistar-rats weighing 200–220 g (aged 8–10 weeks) were purchased from the Pharmacology School of Tehran University of Medical Sciences. All of them were kept in an animal house under standard laboratory conditions at the temperature of 22 ± 1 °C and humidity of 80%, with a typical 12 h light/dark cycle, and allowed to have free access to food and water. All phases of the experiment were approved by the ethics committee of Tehran University of Medical Sciences for the maintenance and application of laboratory animals (IR.TUMS.MEDICINE.REC.1398.593). We do all attempts to minimize animal suffering, and during the experiments to collect brain tissue samples, the animals were euthanized completely by carbon dioxide gas. Then, animal carcasses were disposed of with hospital waste.

The rats were randomly divided into 8 groups (12 rats in each group) as follow:

(I) Control group
(II) Stroke group
(III) Nano micelle group (without drug)
(IV) Pretreated group with BBR (100 mg/kg)
(V) Pretreated group with nano BBR (100 mg/kg)
(VI) Pretreated group with nano BBR (75 mg/kg)
(VII) Pretreated group with nano BBR (50 mg/kg)
(VIII) Pretreated group with nano BBR (25 mg/kg)

The control group hasn’t received any drug and no induction has been made on it. The stroke group induction has been performed but has not received the drug. Also, groups 4–8 were treated orally with BBR and nano-BBR for 14 days with concentrations of 25, 50, 75, 100 mg/kg. For oral administration of berberine hydrochloride, it (approximately 1% of body weight) was dissolved in water [35]. In this study, the vehicle was micelle and the solvent that Berberine and Nano Berberine were diluted in that was water.

### Stroke inducing by BCCAO (bilateral common carotid artery occlusion) model

For the inducing of cerebral ischemia (CI), rats were anesthetized by an intraperitoneal (i.p) injection of 50 mg/kg ketamine and 2–8 mg/kg xylazine. The animals were placed on their back, and their tail and paws were fixed using adhesive tape. A vertical incision (~ 1 cm length) was performed in the neck of each rat, then the bilateral common carotid arteries were revealed, and both carotid arteries were carefully separated from the vagal nerve. Also, it is crucial to avoid any manipulations of the vagal nerve. A 5–0 silk suture loop was made around each carotid artery. Both carotid arteries were occluded for 30 min. Then sixty (60) minutes reperfusion period was initiated. During the reperfusion period, the wounds were sutured and with an anti-septic solution were disinfected, and kept in a separate cage. By finishing the reperfusion period, the rats were decapitated and the whole brain removed for molecular analysis [36–38].

### Biochemical assays

Rats were decapitated under deep anesthesia, the whole brains were removed, and all tissues were stored at − 80 °C for later biochemical assays. The animal’s whole brains were homogenized in buffer TRIS hydrochloride (TRIS HCL), Safety Data Sheet (SDS), Dithiothreitol (DTT), nonyl phenoxy polyethoxyl ethanol (NP40), and glycerol, then centrifuged for 5 min at the room temperature ⁓ 20 to 25 °C (68 to 77 °F) [39], after that, the levels of IL-1β and TNF-α in the supernatant were detected with ELISA kits, also the activity of MDA (as a marker for lipid peroxidation) in the supernatant was measured using the commercial kit according to the manufacturer’s instructions.

### Assay kits were purchased from international and domestic commercial companies (ZellBio GmbH, Ulm, Germany)

#### Statistical analysis

Data analysis was carried out using GraphPad Prism version 7 software. All data were analyzed using one-way analysis of variance (ANOVA) and Tukey post-hoc. Data were presented as Mean ± SEM. Statistically, differences with *p* < 0.05 were recognized as significant.

## Results

### Characterization

**Fig. 1:**
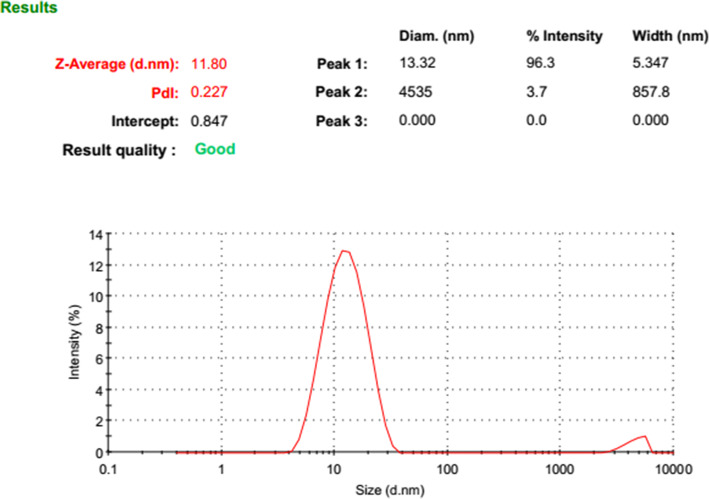
Size distribution diagram based on Intensity

**Fig. 2:**
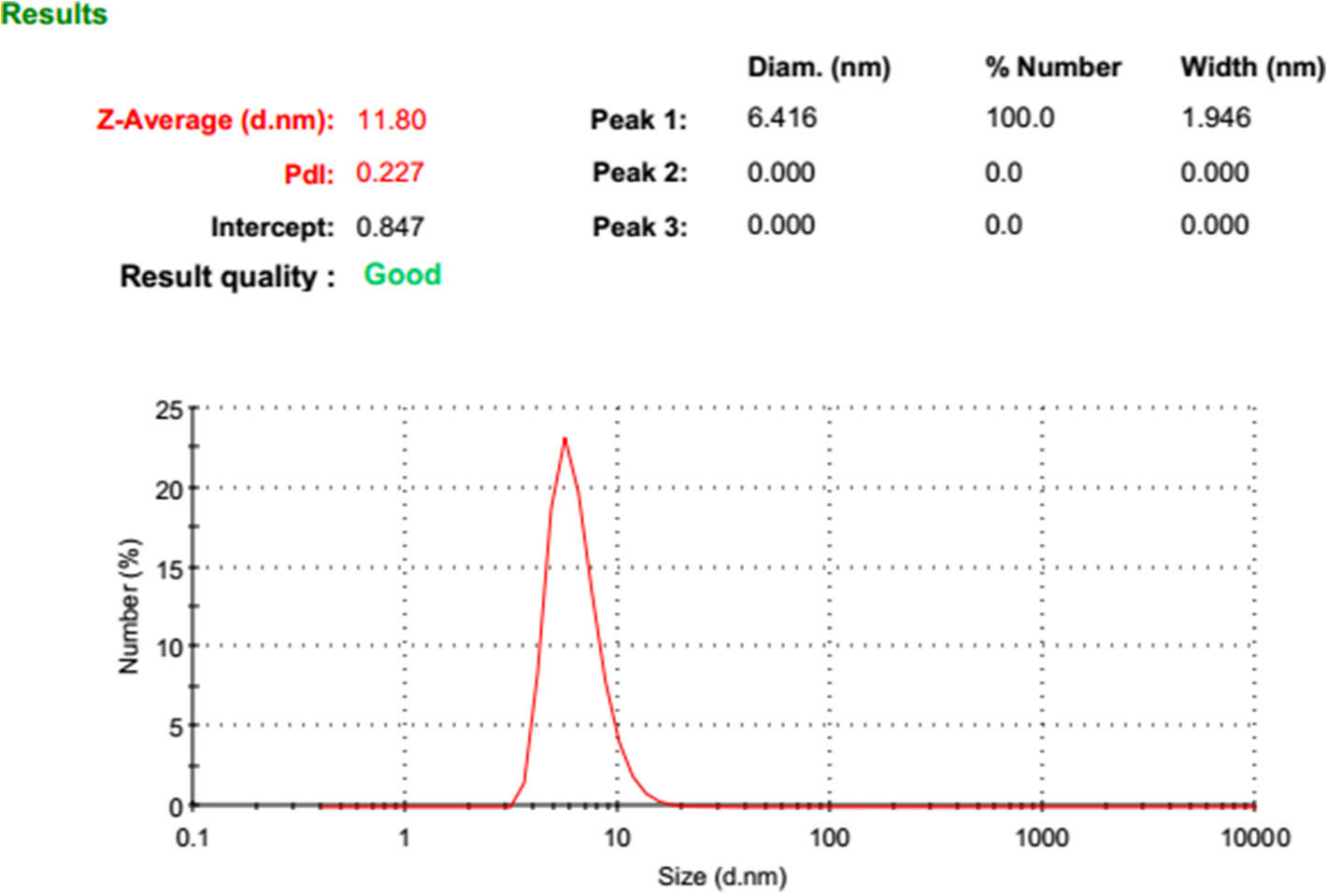
Size distribution diagram based on Number

**Fig. 3:**
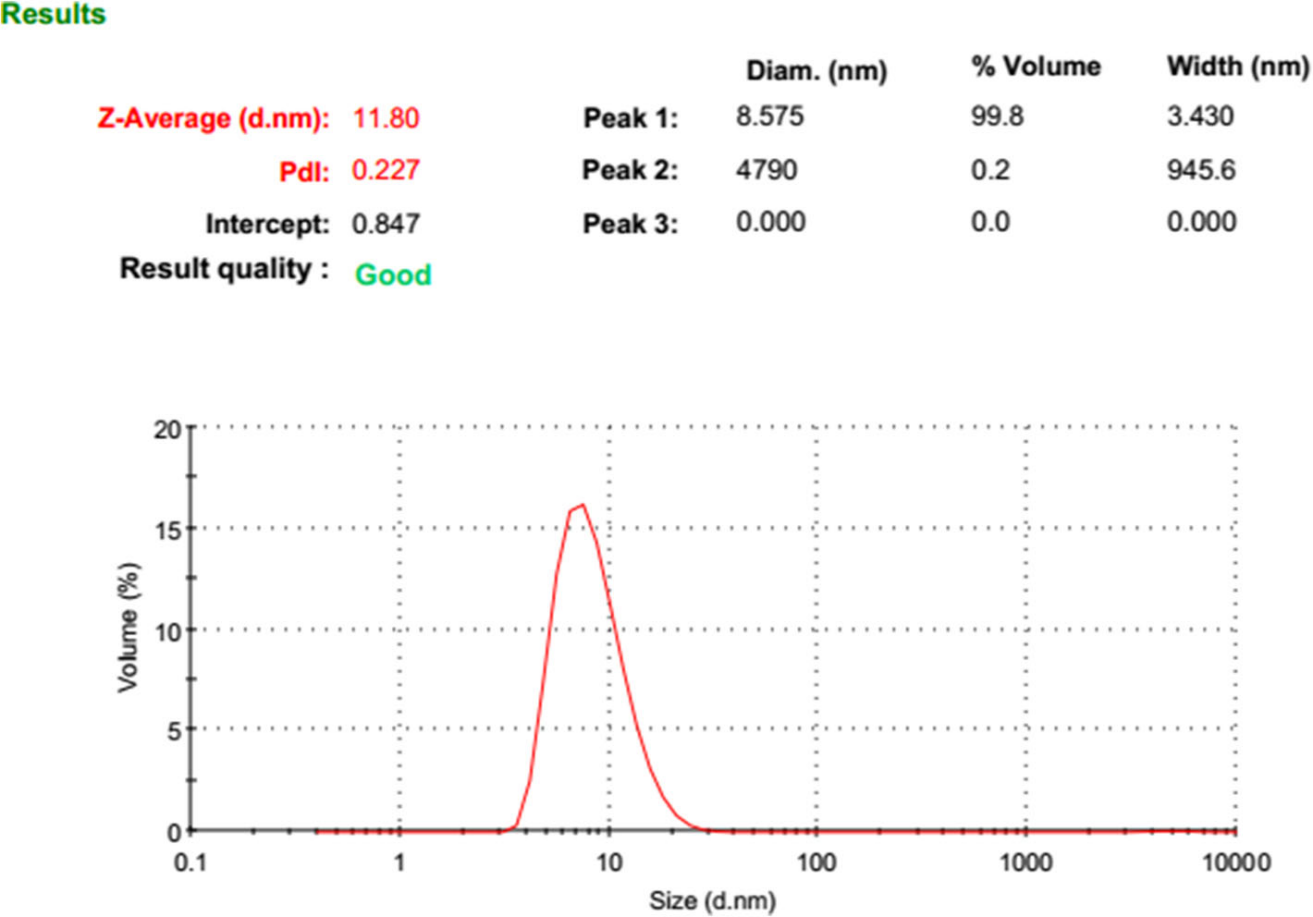
Size distribution diagram based on Volume

**Fig. 4:**
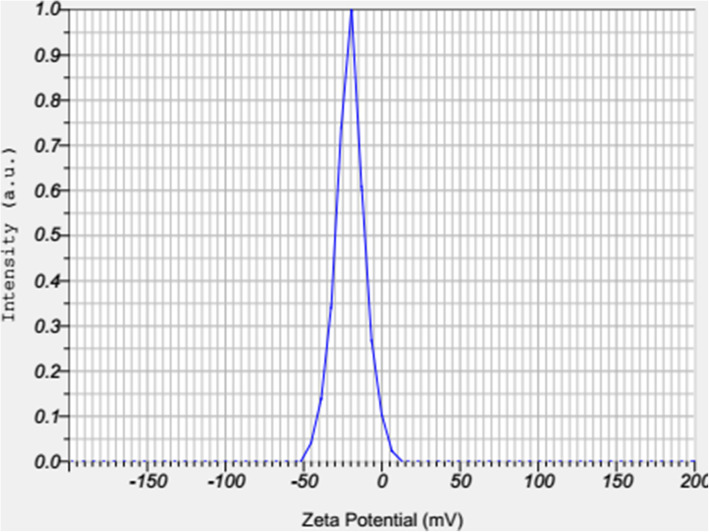
Size distribution Diagram based on Zeta Potential

**Fig. 5:**
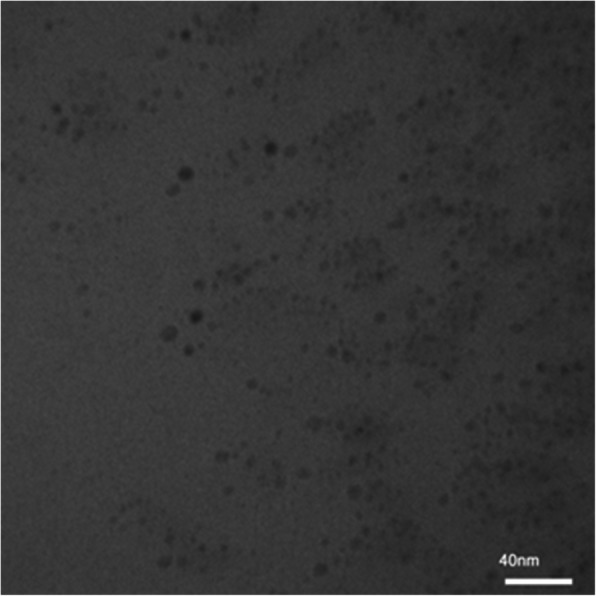
TEM image of BBR Loaded micelle

BBR-loaded micelle formulation successfully was prepared. The dynamic light scattering was used to find out the three of the main parameters of particle size (based on intensity, Number, and  Volume), PDI, and zeta potential of nano micelles (Figs. 1, 2, 3 and 4). DLS results showed an average size about of 12 nm, zeta potential (which represents surface charge) and PDI (which represents the size distribution and system uniformity) were − 22 mv and 0.227, respectively. Also, the size less than 20 nm was confirmed by TEM (Fig. 5). The samples were photographed with a Philips cm300 electron microscope (Philips cm300/ 200 k/ Holland). Diluted nano micelles were dispersed for 20 min and deposited onto a carbon-coated copper grid. After drying, the images were taken, and the morphology of nano micelles using TEM revealed that they have spherical-like shapes.

Encapsulation of BBR into nano micelles was measured by using the centrifugation method, and parameters were calculated according to eq. 1. EE% was calculated as 81%, which suitable for the objective of the current study.

### Drug release study

Percent of drug released from nano micelle formulation in SGF and SIF solutions. Drug release studies in SGF and SIF were evaluated within 24-h sampling, but the maximum drug residence time in the stomach is 2 h, so the results of the first 2 h are important for the survey of drug behavior in SGF.

The level of BBR didn’t fluctuate much in the first 4 h in SGF. However, it was decreased to 71% after 8 h of incubation. The stability of nano micelles was higher in SIF as compared to SGF. In SIF, the concentration of BBR decreased from 97% at 0 h to 86% after 8 h. As the results showed, this time is well enough for nano micelles to pass the stomach and reach the intestine, where they can absorb with different mechanisms.

### Effects of nano BBR-loaded micelle on TNF-α, IL-1ß, and MDA

**Fig. 6:**
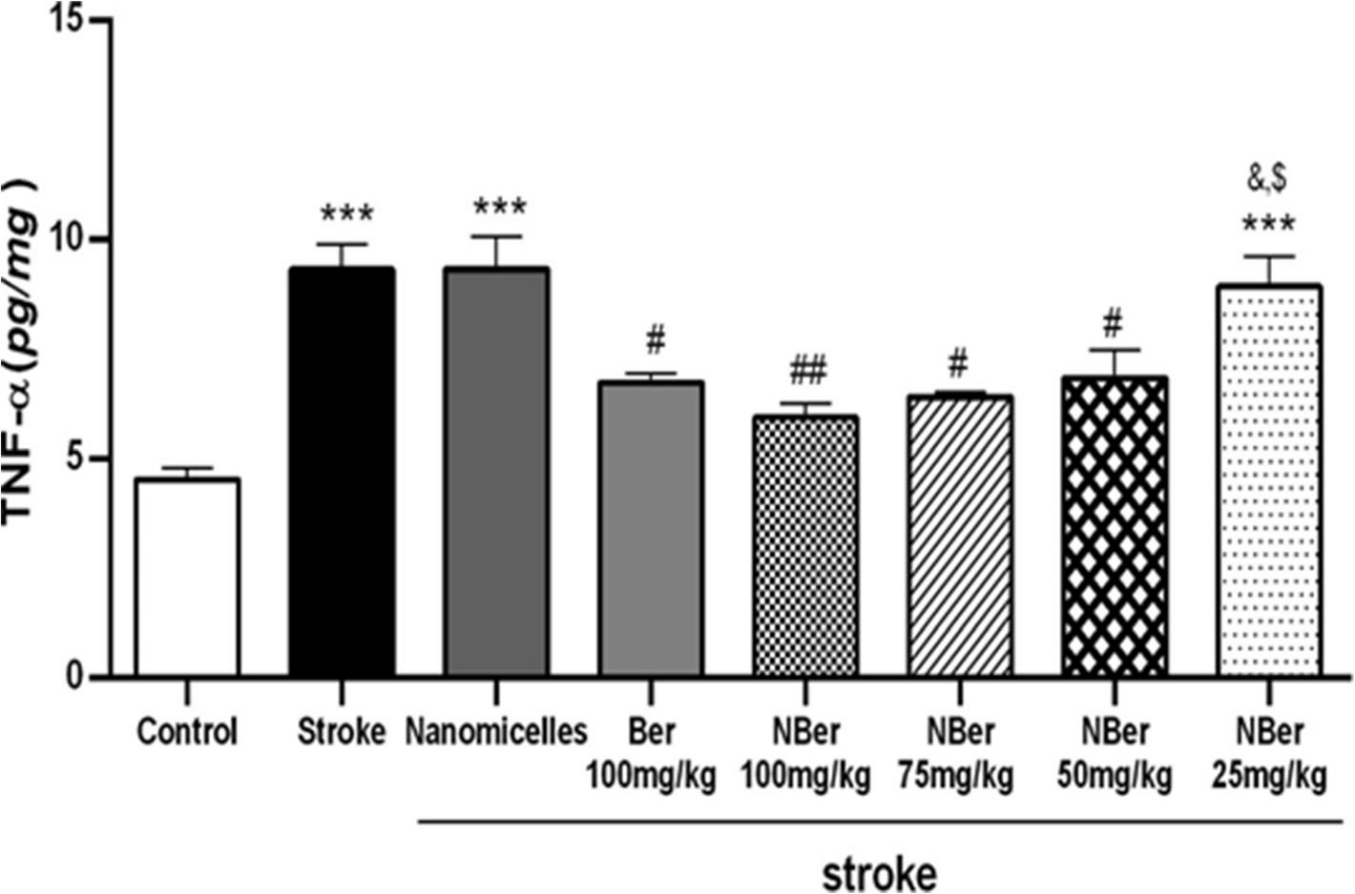
Rate changes of TNF-α in different groups after inducing stroke in rat. Rats were pretreated with BBR (100 mg/kg, oral administration) and BBR-loaded micelle (100, 75, 50, 25 mg/kg) for 14 days before ischemia. ELISA kit was used to detect the level of TNF-α after ischemia. Results show concentrations of BBR 100 mg/kg, and nano BBR 100 mg/kg (*P* < 0.01), 75 and 50 mg/kg (*P* < 0.05) had more reduction in TNF-α level compared to the stroke group

**Fig. 7:**
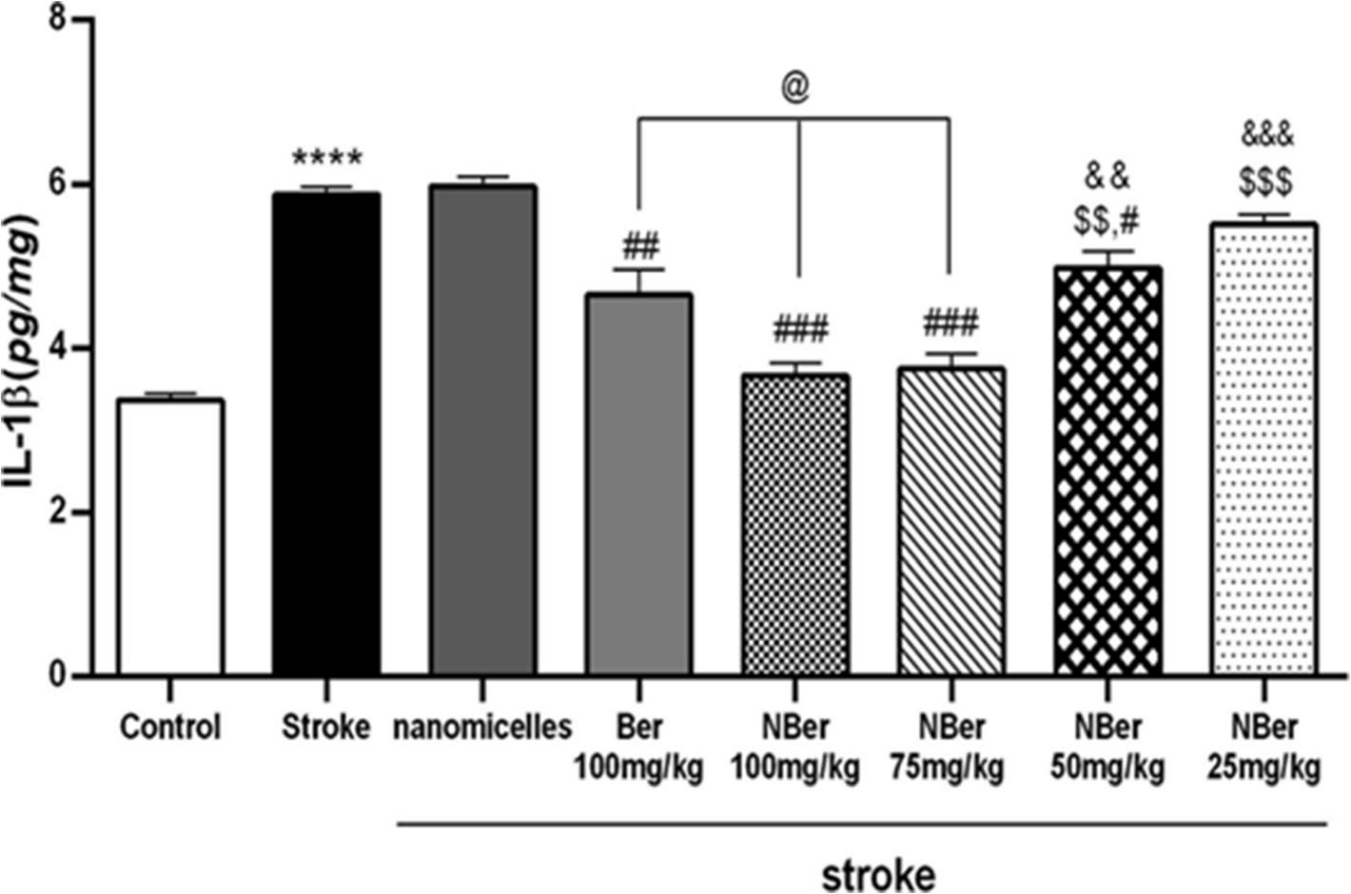
Rate changes of IL-1ß in different groups after inducing stroke in rat. *N* = 8 for each group. *P* < 0.01, *P* < 0.05 compared to stroke group. Data are presented increase the level of IL-1ß in treated group (nano BBR 25 mg/kg) and nano micelle (without drug) group compared to the control group (*p* < 0.001)

**Fig. 8:**
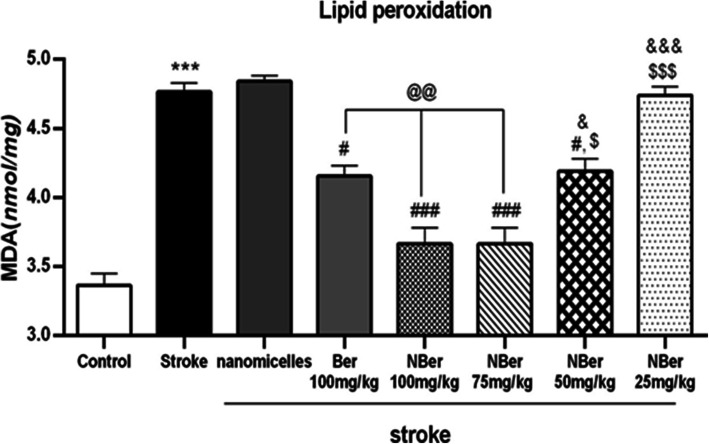
Rate changes of MDA in different groups after inducing stroke in rat

To evaluate the anti-inflammatory effect of berberine, we examined BBR and nano BBR on cytokines and the marker of stress oxidative secreted into brain tissue within cerebral ischemia. Results were shown in (Figs. 6, 7 and 8). After treatment with various concentrations of BBR (100 mg/kg) and nano BBR (25, 50, 75, 100 mg/kg) for 14 days, brain tissues were analyzed by ELISA kits to examine whether the treatments affect the release of inflammatory cytokines. According to the results, the level of TNF-α (from 9 pg/mg in nano micelle group (***) to 6 pg/mg in the treated group (##) with nano BBR concentration of 100 mg/kg), IL_1ß (from 6 pg/mg in nano micelle group to 3.8 pg/mg in the treated group (###) with nano BBR concentration of 100 mg/kg), and MDA (from 4.8 pg/mg in nanomicelle group to 3.6 pg/mg in treated groups with nano BBR concentration (###) of 100 and 75 mg/kg) were reduced in a dose-dependent manner in, indicating the inhibited effect by BBR on the secretion of inflammatory cytokines.

Also, our results show that not only there were no significant differences between the stroke group (***) and nano micelle group (without drug) in every three parameters, but also significant difference was observed between the stroke group (***) and pretreatment groups (@, @@).

It has been accepted that berberine can decrease inflammatory agents-induced IL-1B and TNF-a ensuing inflammation in cerebral ischemia [40]. In the present work, studies have shown that Induction of stroke caused a significant increase in TNF-a, IL-1B, and MDA levels compared to the control group (*p* < 0.001). Also observed increase of cytokines and the marker of oxidative stress in the treated group (nano BBR 25 mg/kg) ($$$, &&&) and nano micelle (without drug) group compared to the control group (p < 0.001).

On the other hand, pretreated groups (@, @@) (BBR 100 mg/kg, nano BBR 100 mg/kg (*p* < 0.01), 75 mg/kg, and 50 mg/kg (*p* < 0.05)) showed a significant reduction in TNF-α, IL-1B, and MDA levels compared to the stroke group.

## Discussion

Different studies reported that several mechanisms have to alleviate cerebral ischemic injury by berberine through its inhibition effects on the level of inflammatory cytokines and oxidative stress including TNF-a, IL-1B, and MDA. Also, several studies have reported similar findings to this one as well, such as Nasseri Maleki et al. reported Berberine might be used as a potential therapeutic agent to reduce inflammation [40]. In addition, Singh and Chopra have shown anti-inflammatory effects of berberine against cerebral ischemia in animal models [41].

The induction of the BCCAO model leads to commence the inflammation pathways by occlusion the blood flow to the brain. In addition, the reperfusion time after an interruption in blood circulation to the brain is a key step to produce the reactive oxygen species (ROS) in the brain cells in particular. Therefore, measurement of TNF-a and IL-1B for assessment of the anti-inflammatory effects, and MDA to assay the effect of drugs in stress oxidative condition in general and on reactive oxygen species (ROS), in particular, could be a usable way [42].

The aim of this study was to emphasize 2 main parameters including preparation and survey of anti-inflammatory effect BBR-loaded micelle nano formulation on cerebral ischemia.

First, BBR-loaded micelles successfully were prepared by the thin-film hydration method. The size of prepared micelles was approximately 12 nm and shows a good distribution. Simoes et al. reported this small size of micelles provides enhanced water solubility of hydrophobic drugs, increased absorption of free drugs, and they release the drug in the target site of the gastrointestinal tract together with a high concentration gradient nearby the epithelium [43]. For these reasons, the small size of micelles has particular importance in oral administration in the drug delivery system. Also, zeta potential shows that the prepared formulation has a negative surface charge that makes it stable against aggregation.

The results presented in this study demonstrated that 81% of BBR molecules successfully entrapped within the core of the micelle. To calculate it, we examined the centrifuge method for the separation of entrapped BBR from the micellar system. Also, we confirmed that the centrifuge method compared to other methods (gel chromatography and dialysis method) is the most appropriate for the separation of free drugs from the micellar system [44]. Drug release studies have shown that BBR was released in a declining trend from nano micelle formulations in SGF and SIF, and they are stable for at least 4 h. Moreover, the BBR release rate from nano micelle formulation was higher in SIF than in SGF.

Second, we compared the effect of BBR and BBR-loaded micelle on TNF-a, IL-1B (inflammations factors), and MDA (marker of oxidative stress) due to inducing cerebral ischemia in the rat. BCCAO model was used to induce stroke into rat’s brain. An increase in inflammation cytokines and MDA have been reported after inducing stroke [45]. In the present study, the results of treated groups (BBR and BBR-loaded micelle) showed a significant decrease in TNF-α, IL-1ß, MDA levels in comparison with the stroke group. This result confirmed by Zhang et al. that reported BBR could be suppressed the activation of proinflammatory cytokines (TNF-α, IL-1ß) after ischemic stroke [9]. This result indicates that the anti-inflammatory properties of berberine and an ameliorate effect against stress oxidative situation that happens due to the BCCAO model. Moreover, this property was further observed in the nano formulation.

Generally, we designed a useful nano formulation, prepared with BBR as drug and micelle as the carrier, BBR-loaded micelle, by thin-film hydration method for reduction of inflammatory effect in cerebral ischemia in the rat. The results of the study suggest that prepared nano formulation possesses improved anti-inflammatory effects more compared to the usual formulation. This effect could be due to the increased aqua solubility in BBR-loaded micelle compared to BBR, resulting in its increased efficiency. Thus, BBR-loaded micelle formulation could be developed as a promising preventive agent on cerebral ischemia.

## Supplementary Information

**Additional file 1.**

**Additional file 2.**

**Additional file 3.**

**Additional file 4.**

## Data Availability

All the necessary data is included in the article. Further data will be shared by request.
