# Supplementary material for: Anti-inflammatory efficacy of Berberine Nanomicelle for improvement of cerebral ischemia: formulation, characterization and evaluation in bilateral common carotid artery occlusion rat model
Source: BMC Pharmacol Toxicol. 2021 Oct 3;22:54. doi: 10.1186/s40360-021-00525-7 (PMC8487542; doi:10.1186/s40360-021-00525-7)
Supplement: Supplementary file 1 — Additional file 1. [file 40360_2021_525_MOESM1_ESM.pdf]

Anti-inflammatory Efficacy of Berberine Nanomicelle for Improvement of Cerebral Ischemia: Formulation, Characterization and Evaluation in Bilateral Common Carotid Artery Occlusion Rat Model

Roza Azadi[1], Seyyedeh Elaheh Mousavi[2,1], Negar Motakef Kazemi[1], Hasan Yousefi-Manesh[2,3], Seyed Mahdi Rezayat[2,3,] , Mahmoud Reza Jaafari[4]

1 Department of Medical Nanotechnology, Faculty of Advanced Sciences and Technology, Tehran Medical Sciences, Islamic Azad University, Tehran, Iran

2 Department of Pharmacology, School of Medicine, Tehran University of Medical Sciences, Tehran, Iran.

3 Department of Medical Nanotechnology, School of Advanced Technologies in Medicine, Tehran University of Medical Sciences, Tehran, Iran.

4 Department of Pharmaceutical Nanotechnology, Mashhad University of Medical Sciences, Mashhad, Iran.

*Corresponding Author.

E-mail address: semousavi@sina.tums.ac.ir

Tel: (+98) 9128479890 (grant No: 98023042949)

PhD Pharmacology Assistant Professor of Pharmacology, School of Medicine, Tehran University of Medical Sciences (TUMS), Tehran, Iran.

Department of Pharmacology, School of Medicine, Tehran University of Medical Sciences, Tehran, Iran.

Acknowledgments

The authors thank the Department of medicine, Faculty of Pharmacology, Tehran University of Medical sciences, Tehran, Iran. (Grant No: 98023042949)

Nanotechnology research center, Pharmaceutical technology institute, Mashhad University of medical sciences, Mashhad, iran.

Authors' contributions

RA and SEM contributed equally to the study conception, design, and preparation of the manuscript. HY, NM, and MRJ contributed to performing the experiments. SEM, MR, and HY supervised the study and contributed to the critical revision of the manuscript. All the authors read and approved the final manuscript.

Funding

This study was financially supported by Experimental Medicine Research Center, Tehran University of Medical Sciences, Tehran, Iran (Grant No. 98023042949)

Availability of data and materials

The datasets used and/or analyzed during the current study available from the corresponding author on reasonable request.

Ethics approval and consent to participate

All the experiments were in accordance with the guidelines of Tehran University of Medical Sciences and experiments were approved by the ethics committee of Tehran University of Medical Sciences.

Consent for publication

Not applicable.

Competing interests

The authors declare that they have no competing interests.

The ARRIVE guidelines

The study was carried out in compliance with ARRIVE guidelines 2.0 .

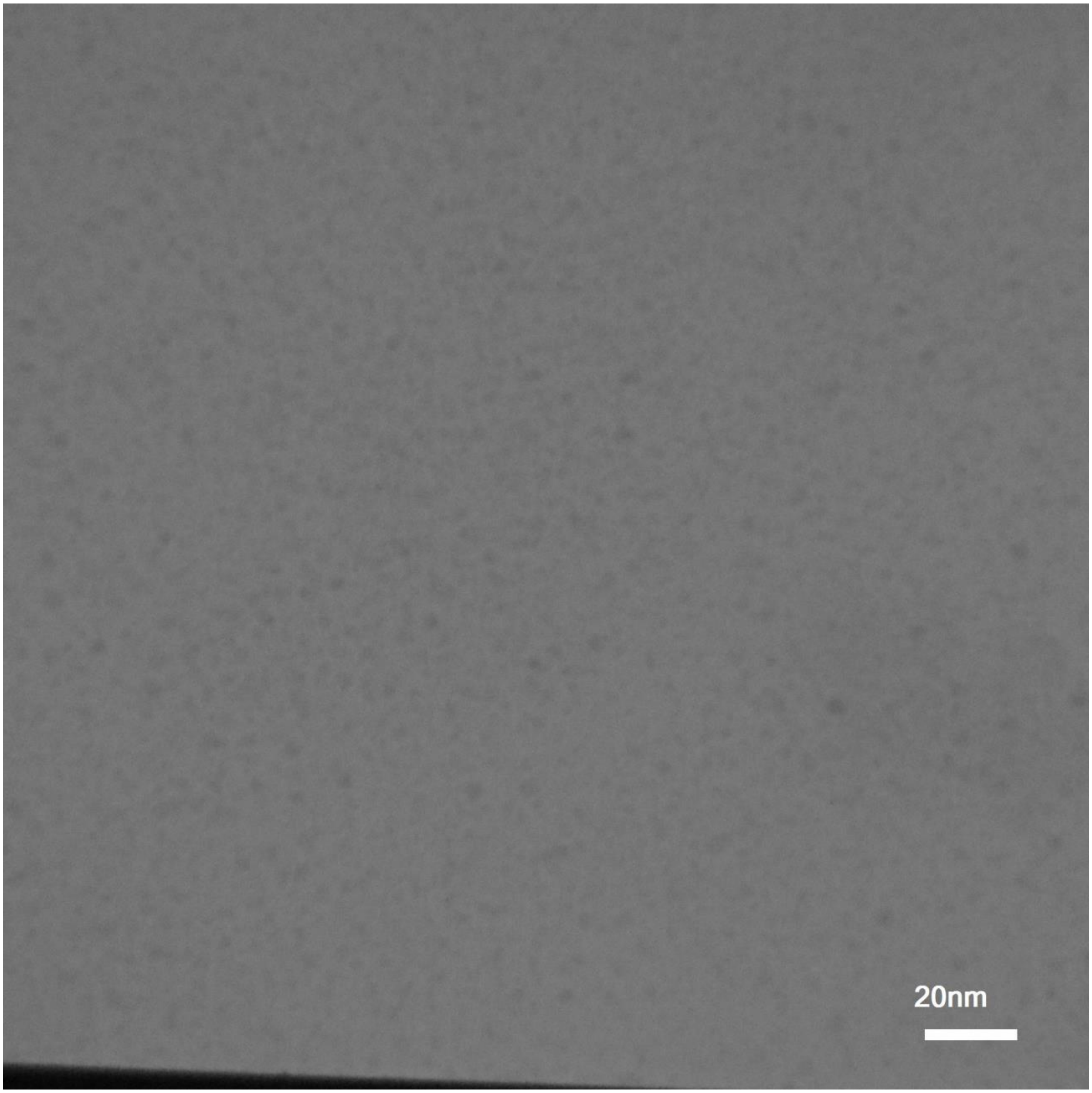
20nm

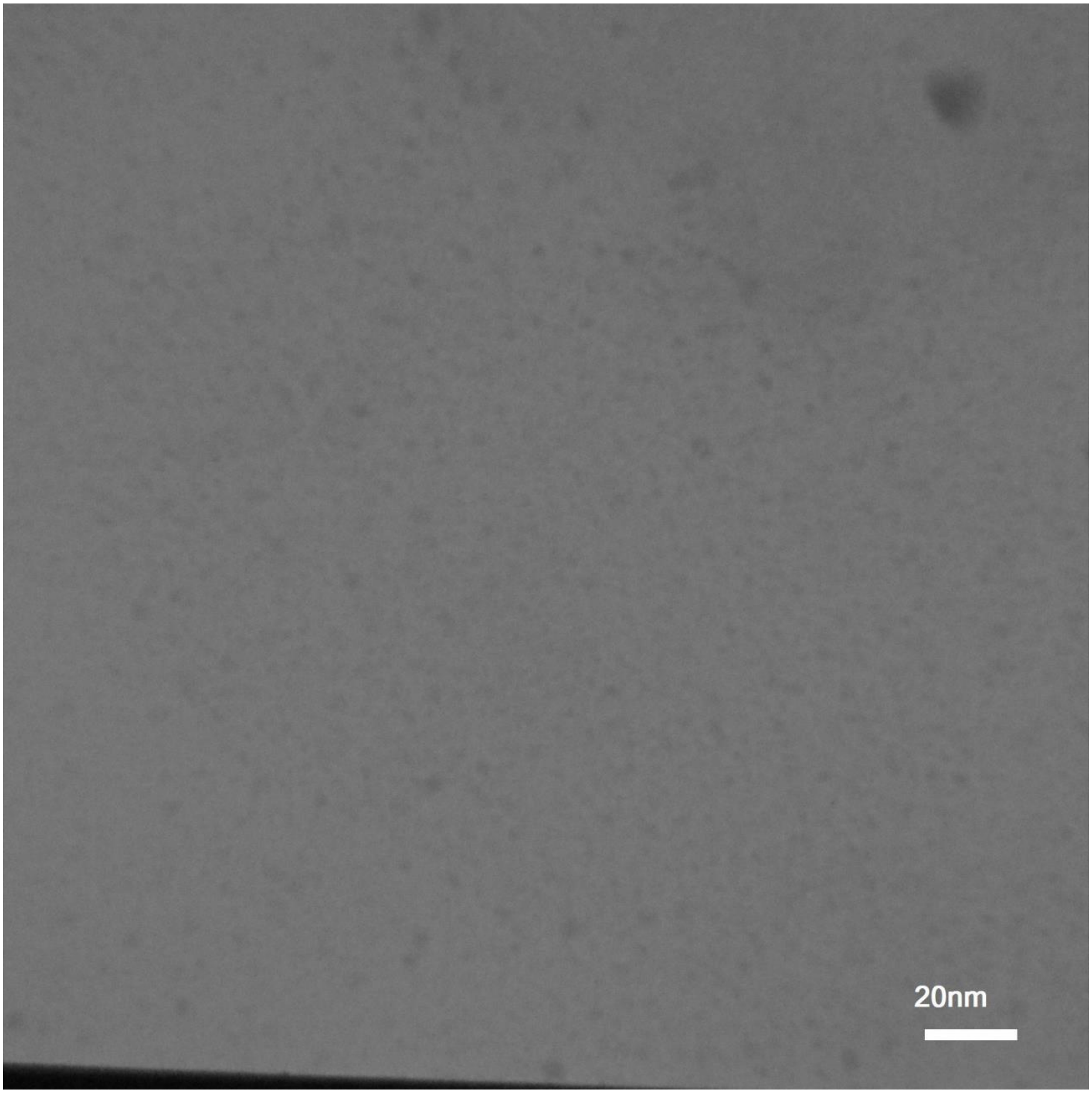
20nm

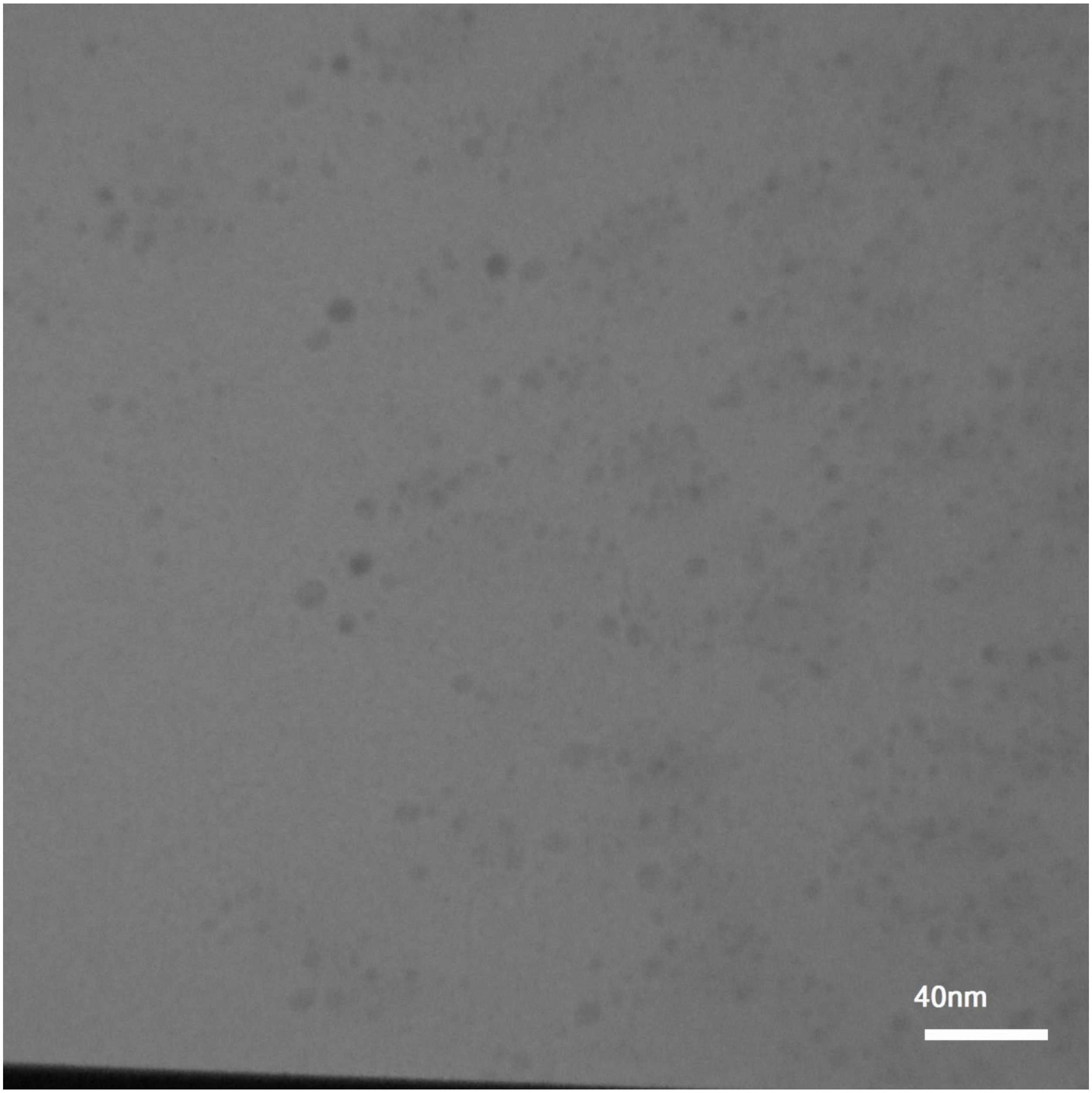
40nm

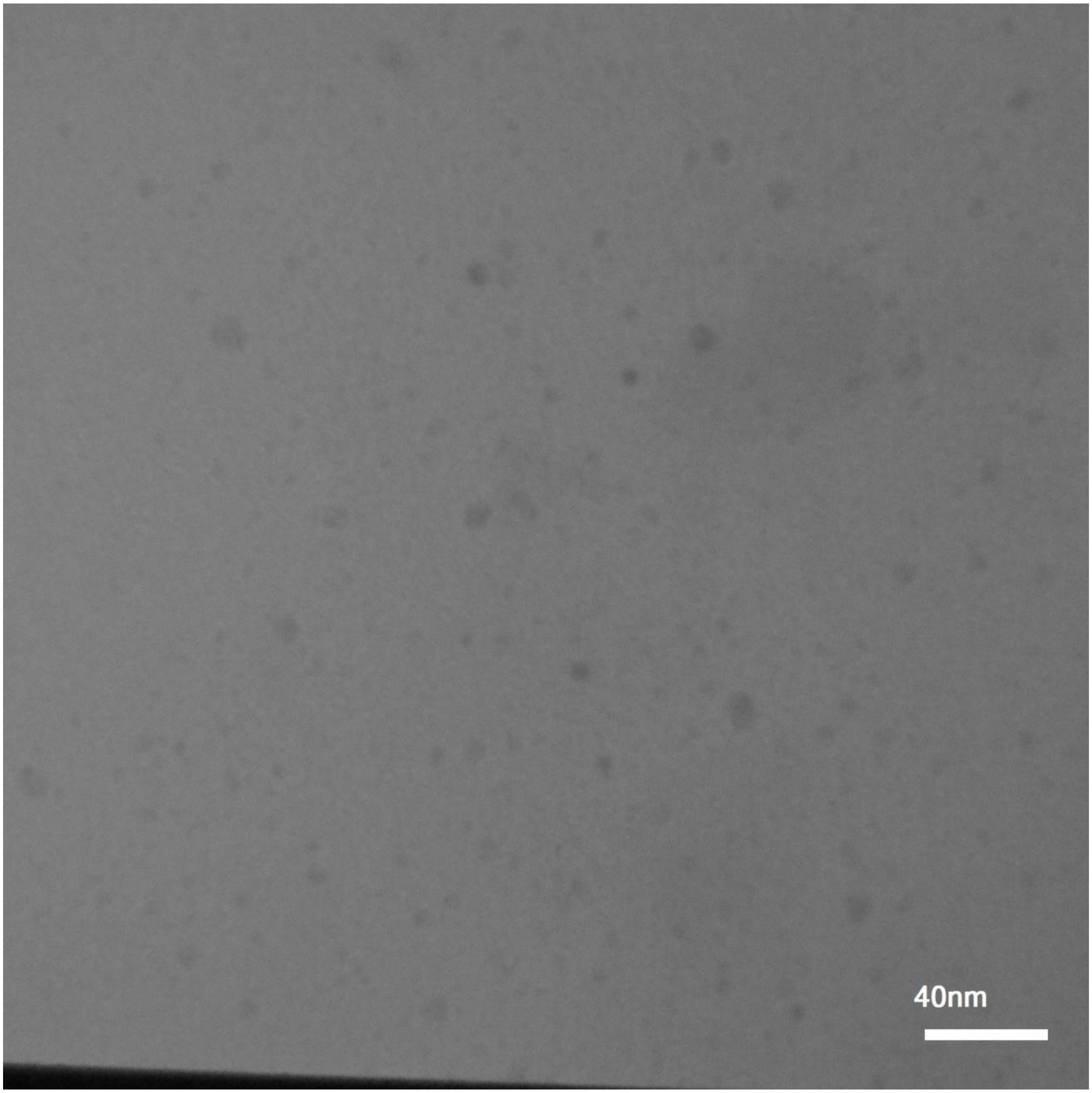
40nm

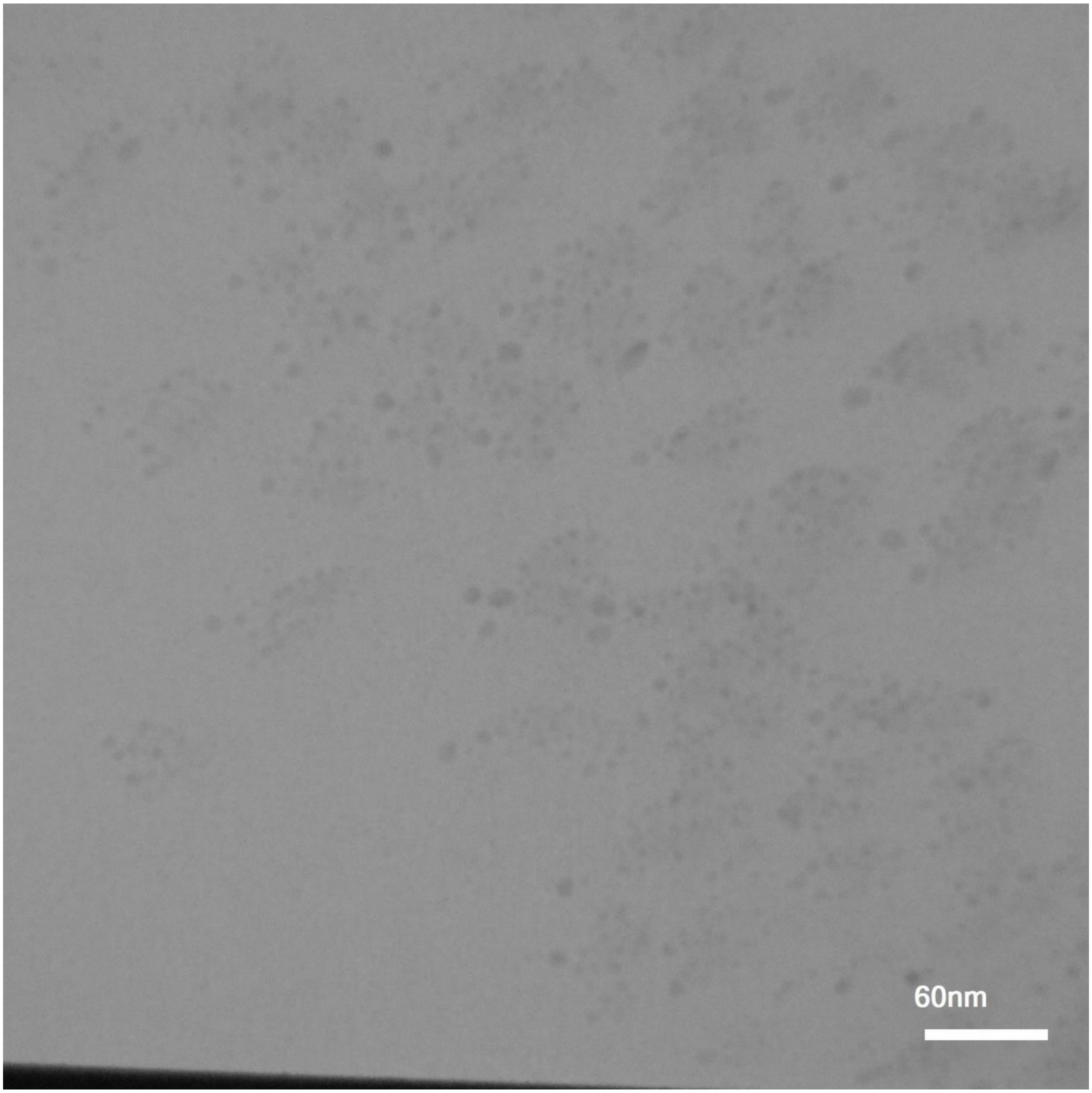
60nm

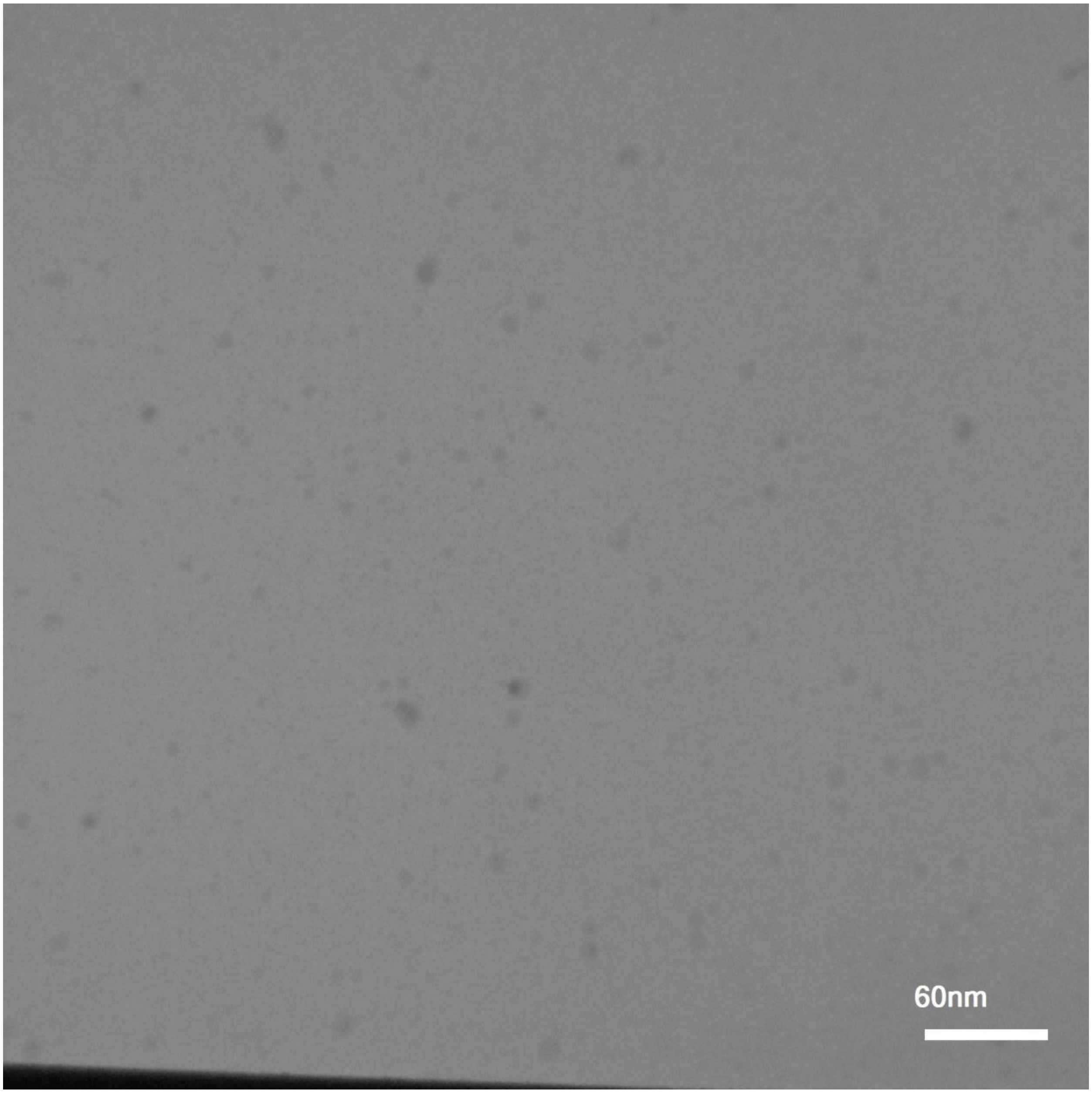
60nm
