# Supplementary material for: Anti-inflammatory efficacy of Berberine Nanomicelle for improvement of cerebral ischemia: formulation, characterization and evaluation in bilateral common carotid artery occlusion rat model
Source: BMC Pharmacol Toxicol. 2021 Oct 3;22:54. doi: 10.1186/s40360-021-00525-7 (PMC8487542; doi:10.1186/s40360-021-00525-7)
Supplement: Supplementary file 3 — Additional file 3. [file 40360_2021_525_MOESM3_ESM.docx]

Drug Release studies in SGF (Simulated Gastric Fluid)

| % Drug Release | Release | Drug Concentration (mg) | time | Time points |
| --- | --- | --- | --- | --- |
| 80.46 | 9.632 | 2.338 | 9 am | 0 |
| 79.61 | 9.53 | 2.44 | 9.30 am | 30 min |
| 79.19 | 9.48 | 2.49 | 10 am | 1 h |
| 77.03 | 9.221 | 2.749 | 11 am | 2 |
| 75.74 | 9.068 | 2.903 | 1 pm | 4 |
| 71.47 | 8.554 | 3.416 | 5 pm | 8 |
| 71.09 | 8.51 | 3.46 | 9 am | 24 |

In SIF (Simulated Intestinal Fluid)

| % Drug Release | Release | Drug Concentration (mg) | time | Time points |
| --- | --- | --- | --- | --- |
| 97.18 | 7.19 | 0.337 | 9 am | 0 |
| 95.43 | 9.64 | 0.547 | 9.30 am | 30 min |
| 92.89 | 9.51 | 0.85 | 10 am | 1 h |
| 92.75 | 9.57 | 1.107 | 11 am | 2 |
| 88.61 | 9.77 | 1.263 | 1 pm | 4 |
| 86.04 | 9.67 | 1.671 | 5 pm | 8 |
| 80.89 | 9.87 | 2.287 | 9 am | 24 |
