# Supplementary material for: Anti-inflammatory efficacy of Berberine Nanomicelle for improvement of cerebral ischemia: formulation, characterization and evaluation in bilateral common carotid artery occlusion rat model
Source: BMC Pharmacol Toxicol. 2021 Oct 3;22:54. doi: 10.1186/s40360-021-00525-7 (PMC8487542; doi:10.1186/s40360-021-00525-7)
Supplement: Supplementary file 4 — Additional file 4. [file 40360_2021_525_MOESM4_ESM.docx]

Formulation ingredients: Surfactant (Deoxicholate) + Drug (BBR) + Water

Surfactant: 49% (w/w): 1.47 gr

Drug: 1% (w/w): 30 mg

Water: 50%: 1.5 ml

$$EE \%=\frac{drug concentration post- filterate drug}{initial concentration of drug} \times100$$

$$EE \%=\frac{11.97 mg- 2.133 mg}{12.208 mg} \times100$$

o.8057×100 = 80.57%
